# Proximal Effects of Blood Flow Restriction on Shoulder Muscle Function and Discomfort During Low-Intensity Exercise

**DOI:** 10.3390/sports13100354

**Published:** 2025-10-04

**Authors:** Junyeop Lee, Kibum Jung, Yongwoo Lee

**Affiliations:** 1 Department of Physical Therapy, Graduate School, Sahmyook University, Seoul 01795, Republic of Korea; youp7824@naver.com (J.L.); kibum921031@gmail.com (K.J.); 2 Department of Physical Therapy, College of Health and Welfare, Sahmyook University, Seoul 01795, Republic of Korea

**Keywords:** blood flow restriction exercise, electromyography, muscle strength, range of motion, shoulder joint, resistance training, rehabilitation

## Abstract

This study aimed to examine the proximal effects of blood flow restriction (BFR) training on shoulder muscle function and subjective discomfort during low-intensity external rotation exercise. Twenty-four healthy adults were randomly assigned to a BFR group or a control group and performed shoulder stabilization exercises with or without BFR. Outcome measures included shoulder external rotation range of motion, maximal isometric strength, muscle endurance, electromyographic activity of the rotator cuff muscles, and perceived discomfort. Both groups demonstrated significant within-group improvements in all outcomes except posterior deltoid and supraspinatus activity (*p* < 0.05). Between-group comparisons showed significantly greater gains in maximal strength and infraspinatus and teres minor activation in the BFR group than in the control group (*p* < 0.05), while discomfort and fatigue scores were also higher in the BFR group (*p* < 0.05). These findings suggest that BFR applied at the proximal upper arm can enhance the strength and activation of key rotator cuff muscles even when cuff placement near the shoulder is limited by anatomy. Proximal BFR may serve as an effective intervention for improving shoulder function when high-intensity exercise is contraindicated, although strategies to minimize discomfort are needed to improve clinical feasibility.

## 1. Introduction

High-intensity resistance training is widely regarded as the most effective method for achieving muscle hypertrophy and strength gains. To induce a significant increase in strength, a minimum of 70% of one-repetition maximum is typically recommended [1]. However, following surgery or in the presence of pain or functional limitations, high-intensity training may not be feasible [2]. Although low-intensity resistance training may enhance muscular endurance and activation, its ability to increase strength remains limited [3].

Blood flow restriction (BFR) training has emerged as an effective alternative to high-intensity resistance training in such situations [4], as applying controlled pressure via a cuff during exercise occludes blood flow, creating venous pooling, increasing metabolic activity, and acidifying the intracellular environment, which in turn activates fast-twitch muscle fibers and replicates the physiological responses typically observed during high-intensity training [5]. For example, previous studies have shown that patients with sarcopenia performed either low-intensity blood flow restriction (BFR) training or conventional high-intensity exercise three times per week for 12 weeks. The results showed that muscle strength increased in both groups, but no significant differences were observed between the groups [6]. Thus, BFR training offers the advantage of reduced injury risk while achieving comparable training outcomes [7]. However, side effects such as numbness, delayed-onset muscle soreness, and compression pain may occur [8].

Recent studies have suggested that BFR training may also affect the proximal muscles near the site of occlusion. These effects may arise through (1) compensatory activation of the proximal muscles due to fatigue in the distal muscles under occlusion [9], (2) paracrine signaling from blood components trapped in the occluded area [10] and (3) systemic release of growth hormones and insulin-like growth factor-1 during BFR [11].

Applying low-intensity external rotation exercises with proximal BFR may contribute to shoulder joint stability [12]. The shoulder, which is structurally optimized for mobility rather than stability, relies heavily on the rotator cuff and surrounding muscles for stabilization. Impairment of these muscles can lead to restricted movement and pain [13]. Weakness in the infraspinatus and teres minor muscles for external rotation can reduce strength and flexibility and contribute to impingement syndromes [14]. Compared to high-intensity exercises, low-intensity training is more effective in selectively activating the rotator cuff, as high-intensity exercises often recruit larger muscles, such as the upper or posterior deltoid [15]. A sustained imbalance in muscle activation may increase the risk of injury, underscoring the importance of targeted training [16].

To date, research on BFR has predominantly focused on its effects on the distal muscles, with limited studies exploring proximal adaptations. This study proposes a combined approach using BFR and low-intensity external rotation exercises for shoulder muscles, where cuff placement is limited by anatomical constraints. Applying low-intensity blood flow restriction (BFR) training to the shoulder muscles may allow healthy adults to minimize the risk of injury associated with high-intensity exercise while achieving injury-prevention benefits through improved muscle activation and strength. In individuals with pain or functional limitations, this approach may help prevent muscle atrophy and facilitate early rehabilitation. This study aimed to investigate the effects of proximal BFR on muscle function and subjective discomfort in healthy adults during shoulder external rotation training.

## 2. Materials and Methods

### 2.1. Study Design

This study utilized a randomized pretest–posttest two-group design over a 4-week intervention period. Participants who met the inclusion criteria were randomly assigned prior to the pretest by drawing papers indicating assignment to either the BFR group (*n* = 12) or the control group (*n* = 12). The independent variable was the type of intervention (low-intensity shoulder external rotation exercise with or without BFR), and the dependent variables included shoulder external rotation range of motion, strength, muscular endurance, electromyographic (EMG) activity, and subjective discomfort and fatigue. All outcome measures used in this study have been previously validated for reliability and are widely used in rehabilitation research. The study was conducted in accordance with the principles of the Declaration of Helsinki. Ethical approval was obtained from the Institutional Review Board of Sahmyook University, Seoul, Republic of Korea (IRB No. SYU 2023-07-012-007; date of approval: 16 November 2023). The trial was subsequently registered with the Clinical Research Information Service (CRIS), Republic of Korea (registration number: KCT0010527). CRIS is a primary registry in the World Health Organization International Clinical Trials Registry Platform (WHO ICTRP), ensuring transparency, accessibility, and adherence to international standards for clinical trial registration.

### 2.2. Participants

Participants were recruited from a metropolitan area. After screening, they were assigned to the BFR group (mean age: 29.1 ± 6.1 years, BMI: 24.5 ± 4.9) or the control group (mean age: 28.4 ± 6.5 years, BMI: 23.4 ± 3.5). The inclusion criteria were as follows: no current pain or injury, absence of orthopedic shoulder disorders, and no shoulder-related exercise in the past 3 months. The exclusion criteria included a history of shoulder dysfunction or injury, limited range of motion [17], vascular diseases, or the use of supplements or medications that could influence muscle metabolism. Participants with recent shoulder exercise experience were excluded to avoid potential confounding effects from muscle adaptations or residual soreness on the study outcomes [12]. All participants provided informed consent after receiving a full explanation of the study procedures.

Sample size estimation was based on a preliminary pilot study that yielded an effect size of 1.74. Using G*Power 3.1 (version 3.1.9.4; Düsseldorf, Germany, 2019) with a significance level of 0.05 and power of 0.95, a sample size of 20 was calculated. Considering a dropout rate of 20%, the final sample comprised 24 participants.

### 2.3. Procedures

**Figure 1:**
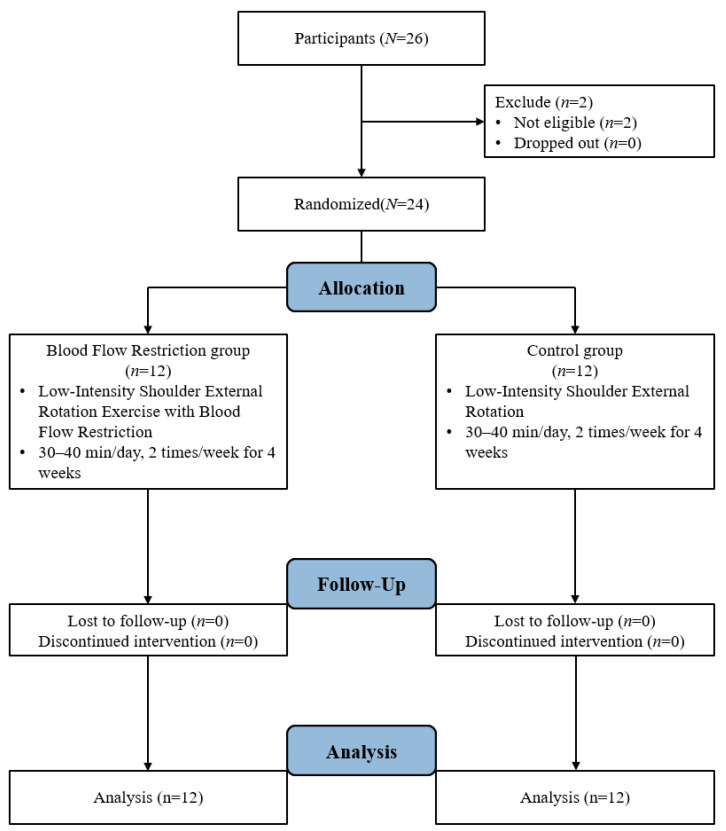
Study Procedure.

Participants were randomly assigned to either the BFR group (*n* = 12) or the control group (*n* = 12). In the BFR group, a cuff was applied to the deltoid tuberosity of the dominant arm using the KATTUS CYCLE 2.0 (KATTUS Co., Tokyo, Japan, 2012), and pressure was set at 80% of the participant’s systolic blood pressure, measured prior to the intervention [18]. Each session began with warm-up exercises, including pendulum motion and assisted active range of motion of shoulder external rotation. The main exercise consisted of shoulder external rotation in both the transverse and sagittal planes: 1 set of 30 repetitions, followed by two sets of 15 repetitions, and a final set performed until fatigue. Each set was separated by 30 s of rest, and 2 min of rest was provided between exercises. Sessions were held twice per week for 4 weeks under one-on-one supervision. Both groups used elastic bands that matched their perceived exertion levels, as determined using the Borg scale (Figure 1).

#### 2.3.1. Shoulder Range of Motion

ROM was assessed using a digital goniometer (iSet Square ver. 1.7; Plaincode, Rosenheim, Germany, 2008). Participants lay supine with their shoulders abducted at 90° while performing active external rotation. Three measurements were performed, and the mean value was used. The intrarater reliability of this method has been reported to be r = 0.79–0.92 [19].

#### 2.3.2. Muscle Strength

Maximal isometric strength during external rotation was measured using a handheld dynamometer (Commander PowerTrack II Dynamometer; J-Tech Medical, Midvale, UT, USA, 2011). Participants lay supine with the shoulder abducted and the elbow flexed at 90°, maintaining the mid-range of external rotation for a 3 s isometric contraction. The average of three trials was used. This method has demonstrated high reliability previously [20]. The dynamometer measurement method has been reported to have a reliability of r = 0.85–0.99 [21].

#### 2.3.3. EMG Activity

A wireless surface EMG system (WAVE PLUS 16ch; COMETA, Bareggio, Italy, 2012) was used to assess the EMG activity of the shoulder muscles during external rotation. The muscles measured included the infraspinatus (2.5 cm below the midpoint of the scapular spine), supraspinatus (supraspinous fossa), teres minor (one-third of the distance between the acromion and inferior angle of the scapula), and posterior deltoid (2.5 cm below the posterior edge of the acromion) [22,23].

The inter-electrode distance was set to 2 cm. To minimize skin impedance, the electrode sites were shaved and cleaned with alcohol swabs [24]. The electrodes were attached horizontally along the direction of the muscle fibers. EMG signal normalization was performed using manual muscle testing to assess the maximum voluntary isometric contraction (MVIC) for each muscle [25].

During the MVIC testing, participants were instructed to exert maximum effort for 5 s, which was repeated three times. A 5 s rest was provided between repetitions and a 1 min rest between different muscles. The elastic band resistance was set according to each participant’s perceived exertion during the pretest, and measurements were initiated at 100% band elongation with no slack [12].

To reliably measure peak muscle activation within a consistent time window, each measurement was repeated three times. For data analysis, a 1 s window centered on the point of peak activity (0.5 s before and after the peak) was averaged. The EMG data were sampled at 2000 Hz and filtered with a bandwidth of 10–350 Hz [26]. The signals were rectified and processed using the root mean square method. All data were analyzed using MyoResearch Master 1.08 XP software (NORAXON, Scottsdale, AZ, USA, 2017). The EMG activity of each muscle was normalized as a percentage of the MVIC.

#### 2.3.4. Muscle Endurance

Endurance was assessed with participants standing, their elbows flexed at 90°, and performing external rotation with an elastic band in the transverse plane. Participants were trained to perform 20 repetitions in 60 s using a metronome. They continued the motion until failure, which was defined as an inability to maintain the form. The goniometers ensured consistent arm positions and band lengths. The test began with the band under 100% tension (without slack) [12].

#### 2.3.5. Subjective Discomfort and Fatigue

Discomfort and fatigue were measured using a visual analog scale ranging from 1 (no discomfort/fatigue) to 10 (extreme discomfort/fatigue). Participants rated discomfort immediately after the final exercise session and rated fatigue after completing the third set of the second exercise during the final session [27].

### 2.4. Statistical Analyses

Data were analyzed using IBM SPSS Statistics (version 22.0; IBM Corp., Armonk, NY, USA, 2014). The Shapiro–Wilk test confirmed normality for all data except for supraspinatus EMG activity, which still met the distribution criteria (skewness = 2.034, kurtosis = 4.375). Homogeneity between groups was verified using chi-square and independent *t*-tests. Paired *t*-tests were used to assess pre-post changes within the groups, and independent *t*-tests were used to compare differences between the groups. Statistical significance was set at *p* < 0.05.

## 3. Results

The general characteristics of the participants are shown in Table 1 and their homogeneity is shown in Table 2. The results before and after the intervention are summarized in Table 3. A survey of the participants’ discomfort is presented in Table 4.

### 3.1. Muscle Endurance

Both the BFR and control groups demonstrated significant improvements in shoulder external rotation endurance after the intervention (*p* < 0.05). However, there was no significant difference in the magnitude of change between the groups.

### 3.2. Maximal Strength of External Rotation

Both groups showed significant increases in the maximal isometric strength of external rotation after the intervention (*p* < 0.05), with the BFR group demonstrating a significantly greater improvement than the control group (*p* < 0.05).

Range of motion of external rotation: Both groups exhibited significant post-intervention improvements in shoulder external rotation ROM (*p* < 0.05). However, no significant between-group differences in the change scores were observed.

### 3.3. EMG Activity

Posterior deltoid: Both groups showed significant increases in muscle activation after the intervention (*p* < 0.05), but there were no significant differences in the magnitude of change between groups. Supraspinatus: No significant within- or between-group differences were observed. Infraspinatus and teres minor: Both groups showed significant increases in muscle activity after the intervention. The BFR group demonstrated significantly greater activation gain than the control group (*p* < 0.05).

### 3.4. Subjective Discomfort

Following the intervention, the BFR group reported significantly higher levels of discomfort and fatigue than the control group (*p* < 0.05).

## 4. Discussion

This study investigated the effects of low-intensity shoulder external rotation training combined with BFR on muscle endurance, maximal strength, ROM, EMG activity, and perceived discomfort. Participants in the BFR group exhibited significantly greater increases in maximal strength and activity of the infraspinatus and teres minor, while also experiencing higher levels of discomfort and fatigue than those in the control group.

Previous research has shown that applying BFR cuffs to the proximal humerus during exercises, such as a bench press or shoulder press, significantly enhances strength gains in the pectoral and deltoid muscles, respectively. These findings align with the results of the present study, in which BFR induced superior improvements in maximal strength and muscle activation. These effects may be related to the systemic responses induced by BFR training, such as increased growth hormone secretion and subsequent insulin-like growth factor-1 (IGF-1) synthesis, which could contribute to strength enhancement [11]. However, it should be noted that this study did not directly measure these biochemical markers, and therefore, these explanations remain hypothetical. Additionally, fatigue in the distal muscles under occlusion may increase the recruitment of proximal muscles, and altered cellular environments in distal tissues (hypoxia and metabolite accumulation) could potentially exert paracrine effects on proximal muscles [28]. However, these mechanisms are also considered speculative.

The volume of growth hormone released is believed to vary with exercise type, with large muscle groups or full-body movements eliciting higher hormonal responses than isolated upper-limb exercises. Other studies have suggested that, to maximize the proximal effects of BFR during small muscle group exercises, a longer duration of occlusion may be required to induce sufficient fatigue and hormonal responses [29]. Therefore, future protocols may benefit from extended occlusion times; however, discomfort must be carefully managed to maintain patient compliance.

The absence of changes in supraspinatus activation can be attributed to two factors. First, the primary function of the supraspinatus is during shoulder abduction, and while it contributes to external rotation, its activation predominantly occurs in the early phase of the movement [30]. Thus, the low-intensity external rotation exercises performed in this study may not have provided sufficient stimulus to elicit a measurable increase in supraspinatus activity. Second, anatomically, the supraspinatus lies beneath the upper trapezius [31], which may have limited the accuracy of its assessment using surface electromyography.

Both groups showed significant post-intervention gains in muscular endurance. Although no between-group differences were observed, the BFR group showed an improvement in endurance by 43.86%, whereas the control group showed an improvement in endurance by 35.14%. These results suggest that BFR may enhance endurance, but a longer intervention duration, possibly exceeding 6 weeks, may be necessary to observe significant between-group effects.

Shoulder ROM also increased significantly in both groups, without any between-group differences. Previous studies have indicated that repetitive external rotation exercises using elastic bands help maintain humeral head centralization within the glenoid, thus facilitating posterior roll and anterior glide [32]. These exercises also enhance the activation of the infraspinatus and teres minor muscles. Therefore, the gains in ROM observed in this study were likely due to repetitive motion rather than BFR itself.

Subjective discomfort and fatigue scores were significantly higher in the BFR group. Previous studies have reported that up to 92% of participants experience discomfort during BFR, primarily due to numbness [33]. Rapid accumulation of lactate under occlusion may be a key factor. One study found that lactate levels increased by 1.9 mmol/L more in the BFR walking group than in the control group [34]. In addition, the physical presence and pressure of the cuff may contribute to psychological discomfort [35]. In contrast, low lactate accumulation during standard low-intensity training likely explains the relatively low levels of fatigue reported in the control group [36]. Factors that appear to influence discomfort are the applied pressure and cuff width [37]. Therefore, blood flow restriction should be applied using individualized pressures, and the use of narrower cuffs may help reduce discomfort.

The study has several limitations. First, surface EMG is limited in its ability to accurately isolate deep muscle activity. Second, the sample size was relatively small and restricted to healthy young adults (with males outnumbering females), which limits the generalizability of the findings to clinical populations where BFR may be most relevant, such as patients with shoulder injuries or those recovering from surgery. Third, the 4-week intervention period may have been insufficient to fully assess changes in muscle endurance and range of motion, indicating that a longer intervention may be necessary.

Future studies should consider using intramuscular EMG to assess deep muscles, including larger and more diverse samples, and implementing stricter control of activities outside the training sessions. Additionally, evaluating changes in muscle size using ultrasound could provide a more quantitative assessment of the effects of low-intensity BFR training.

## 5. Conclusions

This study investigated the proximal effects of blood flow restriction (BFR) training on shoulder muscle function and subjective discomfort during external rotation exercises. The results demonstrated that BFR training significantly enhanced the strength and activation of the infraspinatus and teres minor muscles, although it was also associated with greater discomfort and fatigue compared to conventional low-intensity exercise. These findings suggest that proximal BFR training can serve as an effective intervention for improving shoulder function in contexts where high-intensity exercise is limited, such as post-surgical rehabilitation, older adults, or injury prevention in healthy individuals. However, strategies to reduce discomfort are necessary to enhance exercise feasibility and adherence.

## Figures and Tables

**Table 1:** General characteristics of the participants.

	Experimental Group (*n* = 12)	Control Group (*n* = 12)	χ^2^/t(*p*)
Sex (male/female)	9/3	8/4	0.202 ^c^ (0.653)
Measurement area (R/L)	12/0	11/1	1.403 (0.307)
Age (year)	29.16 ^a^ ± 6.11	28.41 ± 6.51	−0.705 ^b^ (0.481)
Height [25]	170.33 ± 9.06	171.50 ± 8.66	−0.231 (0.817)
Weight (kg)	72.41 ± 20.40	59.91 ± 15.89	−0.029 (0.977)
BMI	24.07 ± 3.19	23.36 ± 1.82	−0.05 (0.506)

Note. ^a^ Mean ± standard deviation; ^b^ Independent samples *t*-test; ^c^ Chi-square test; BMI: Body mass index.

**Table 2:** Homogeneity test for each measurement item of the study participants.

	Experimental Group (*n* = 12)	Control Group (*n* = 12)	t/(*p*) ^b^
Muscular endurance (time)	50.16 ± 15.83 ^a^	50.50 ± 10.36	−0.202 (0.840) ^b^
Maximum strength (N)	47.50 ± 11.80	52.58 ± 15.10	−0.954 (0.340)
Shoulder external rotation range of motion (°)	81.00 ± 4.36	79.58 ± 3.94	−0.698 (0.485)
Posterior deltoid (% MVIC)	2.99 ± 1.64	3.46 ± 2.45	−0.058 (0.954)
Supraspinatus (% MVIC)	4.32 ± 1.18	5.60 ± 4.08	−0.346 (0.729)
Infraspinatus (% MVIC)	38.37 ± 10.24	45.83 ± 10.96	−1.443 (0.149)
Teres minor (% MVIC)	31.32 ± 14.76	36.50 ± 11.12	−1.328 (0.184)

Note. ^a^ Mean ± standard deviation; ^b^ Independent samples *t*-test; %MVIC: % maximal voluntary isometric contraction.

**Table 3:** Study results.

		Experimental Group (*n* = 12)	Control Group (*n* = 12)	t(*p*) ^b^	Effect Sizes
Muscular endurance (time)	Pre	50.16 ± 15.83 ^a^	50.50 ± 10.36		
	Post	71.91 ± 2.86	68.25 ± 2.61		
	Pre-Post	21.50 ± 10.56	17.66 ± 8.82	1.007 (0.325) ^b^	0.39
	t(*p*)	−7.130 (0.000) *	−5.219 (0.000) *		
Maximum strength (N)	Pre	47.50 ± 11.80	52.58 ± 15.10		
	Post	58.08 ± 10.29	58.33 ± 14.06		
	Pre-Post	10.58 ± 5.88	5.75 ± 3.81	2.387 (0.026) *	0.97
	t(*p*)	−6.230 (0.000) *	−5.219 (0.000) *		
Shoulder external rotation Range of motion (º)	Pre	81.00 ± 4.36	79.58 ± 3.94		
	Post	85.83 ± 2.65	84.00 ± 4.13		
	Pre-Post	4.83 ± 3.71	4.41 ± 3.65	0.277 (0.784)	0.11
	t(*p*)	−4.509 (0.001) *	−4.186 (0.002) *		
Posterior deltoid (% MVIC)	Pre	2.99 ± 5.54	3.46 ± 2.45		
	Post	5.54 ± 3.96	3.90 ± 2.25		
	Pre-Post	2.55 ± 4.11	0.69 ± 2.70	1.303 (0.208)	0.53
	t(*p*)	−2.159 (0.054)	−1.820 (0.096)		
Supra spinatus (% MVIC)	Pre	4.32 ± 1.81	5.60 ± 4.08		
	Post	5.40 ± 2.63	4.70 ± 1.40		
	Pre-Post	0.76 ± 2.09	−0.38 ± 5.33	0.692 (0.496)	0.28
	t(*p*)	−1.335 (0.209)	0.643 (0.533)		
Infra spinatus (% MVIC)	Pre	38.37 ± 10.24	45.83 ± 10.96		
	Post	64.93 ± 11.07	60.07 ± 12.72		
	Pre-Post	25.56 ± 12.61	14.15 ± 11.54	2.514 (0.020) *	0.94
	t(*p*)	−7.294 (0.000) *	−4.246 (0.001) *		
Teres minor (% MVIC)	Pre	31.32 ± 14.76	36.50 ± 11.12		
	Post	58.42 ± 16.20	51.61 ± 13.19		
	Pre-Post	27.10 ± 12.61	14.74 ± 11.63	2.703 (0.013) *	1.02
	t(*p*)	−8.726 (0.000) *	−4.316 (0.001) *		

Note. ^a^ Mean ± standard deviation; ^b^ Independent samples *t*-test; %MVIC: % maximal voluntary isometric contraction; * *p* < 0.05.

**Table 4:** Survey results of participants’ discomfort.

Category		Score		t(*p*) ^b^
VAS		Experimental group (*n* = 12)	Control group (*n* = 12)	
1	How much did you feel overall discomfort when you exercise?	44.58 ± 15.58 ^a^	10.42 ± 0.78	6.786 (0.000 *)
2	How hard was it for you after 3 sets?	64.17 ± 11.64	54.16 ± 10.83	2.178 (0.040 *)

Note. ^a^ Mean ± standard deviation; ^b^ Independent samples *t*-test; VAS: Visual analog scale; * *p* < 0.05.

## Data Availability

The original contributions presented in the study are included in the article, further inquiries can be directed to the corresponding author/s.
